# The Molecular Determinants of Small-Molecule Ligand Binding at P2X Receptors

**DOI:** 10.3389/fphar.2018.00058

**Published:** 2018-02-02

**Authors:** Gaia Pasqualetto, Andrea Brancale, Mark T. Young

**Affiliations:** ^1^School of Pharmacy and Pharmaceutical Sciences, Cardiff University, Cardiff, United Kingdom; ^2^School of Biosciences, Cardiff University, Cardiff, United Kingdom

**Keywords:** P2X, ligand binding, molecular modeling, structure-function, ion channel

## Abstract

P2X receptors are trimeric eukaryotic ATP-gated cation channels. Extracellular ATP—their physiological ligand—is released as a neurotransmitter and in conditions of cell damage such as inflammation, and substantial evidence implicates P2X receptors in diseases including neuropathic pain, cancer, and arthritis. In 2009, the first P2X crystal structure, *Danio rerio* P2X4 in the *apo*- state, was published, and this was followed in 2012 by the ATP-bound structure. These structures transformed our understanding of the conformational changes induced by ATP binding and the mechanism of ligand specificity, and enabled homology modeling of mammalian P2X receptors for ligand docking and rational design of receptor modulators. P2X receptors are attractive drug targets, and a wide array of potent, subtype-selective modulators (mostly antagonists) have been developed. In 2016, crystal structures of human P2X3 in complex with the competitive antagonists TNP-ATP and A-317491, and *Ailuropoda melanoleuca* P2X7 in complex with a series of allosteric antagonists were published, giving fascinating insights into the mechanism of channel antagonism. In this article we not only summarize current understanding of small-molecule modulator binding at P2X receptors, but also use this information in combination with previously published structure-function data and molecular docking experiments, to hypothesize a role for the dorsal fin loop region in differential ATP potency, and describe novel, testable binding conformations for both the semi-selective synthetic P2X7 agonist 2′-(3′)-O-(4-benzoyl)benzoyl ATP (BzATP), and the P2X4-selective positive allosteric modulator ivermectin. We find that the distal benzoyl group of BzATP lies in close proximity to Lys-127, a residue previously implicated in BzATP binding to P2X7, potentially explaining the increased potency of BzATP at rat P2X7 receptors. We also present molecular docking of ivermectin to rat P2X4 receptors, illustrating a plausible binding conformation between the first and second transmembrane domains which not only tallies with previous mutagenesis studies, but would also likely have the effect of stabilizing the open channel structure, consistent with the mode of action of this positive allosteric modulator. From our docking simulations and analysis of sequence homology we propose a series of mutations likely to confer ivermectin sensitivity to human P2X1.

## Introduction

In recent years, our understanding of the relationship between the structure and the function of the ATP-gated P2X receptor family of ion channels has been transformed by a series of crystal structures, from the first structure of a P2X receptor, that of *Danio rerio* P2X4.1 (zfP2X4) in the *apo*-state, published in 2009 (Kawate et al., 2009), via structures of zfP2X4 bound to ATP (Hattori and Gouaux, 2012), a Gulf Coast tick (*Amblyomma maculatum*) P2X structure (Kasuya et al., 2016), human P2X3 in the *apo*-, ATP- and antagonist-bound states (Mansoor et al., 2016), zfP2X4 bound to the partial agonist CTP (Kasuya et al., 2017a), to the most recently determined structures of giant panda (*Ailuropoda melanoleuca*) P2X7 (Karasawa and Kawate, 2016) and chicken P2X7 (Kasuya et al., 2017b). These impressive achievements, along with their enabling of the interpretation of a large body of prior mutagenesis data (reviewed in Chataigneau et al., 2013; Jiang et al., 2013; Alves et al., 2014; Samways et al., 2014; Grimes and Young, 2015; Habermacher et al., 2016; Kawate, 2017), have led to significant breakthroughs in our understanding of channel architecture, ligand binding, and the mechanisms of channel opening, desensitization and both orthosteric and allosteric antagonism. In addition, the availability of structural data has allowed for the construction and testing of molecular models of those human receptors which still lack direct high-resolution structural data (Alves et al., 2014; Ahmadi et al., 2015; Caseley et al., 2015, 2016; Farmer et al., 2015; Fryatt et al., 2016), paving the way for mutational analysis to elucidate antagonist binding sites (Farmer et al., 2015; Allsopp et al., 2017), and structure-aided drug design (Ahmadi et al., 2015; Caseley et al., 2015, 2016).

P2X receptors play wide-ranging physiological roles and are important in health and disease progression (North, 2016); for this reason they have been significant targets for the pharmaceutical industry (Burnstock, 2017). The functional unit of the P2X receptor is a trimer, and seven P2X receptor subtypes are found in mammals (numbered P2X1-P2X7), which can form a variety of both homo-and hetero-trimeric receptors, which display subtly different pharmacological properties and tissue distribution (North, 2016). Developing potent, subtype-selective P2X receptor modulators has been a slow process, but several such compounds are now available (Bartlett et al., 2014; Burnstock, 2017), including the P2X7 antagonists CE-224,535 (Stock et al., 2012) and AZD9056 (McInnes et al., 2007; Keystone et al., 2012; Eser et al., 2015), and the P2X3 antagonist AF-219 (Abdulqawi et al., 2015), which have been used in clinical trials for rheumatoid arthritis, Crohn's disease and chronic cough, respectively. The majority of developed compounds are antagonists; at present there are no truly subtype-selective P2X receptor agonists available, although α,β-methylene ATP shows selectivity for P2X1 and P2X3 receptors, and BzATP is partially selective for P2X7 receptors (North, 2002). One compound, the anti-helminthic drug ivermectin, has also been reported to be a selective (in terms of P2X receptors) positive allosteric modulator of P2X4 receptors (Khakh et al., 1999). For the majority of known P2X modulators, their binding sites have not yet been elucidated. However, the recent crystal structures of human P2X3 in complex with the orthosteric antagonists A-317491 and trinitrophenyl-ATP (TNP-ATP) (Mansoor et al., 2016), chicken P2X7 in complex with TNP-ATP (Kasuya et al., 2017b), and panda P2X7 in complex with a series of allosteric antagonists including A-740003 (Karasawa and Kawate, 2016) have given valuable insight into the binding modes and mechanism of antagonism of these compounds.

In this article our aim is to describe our current understanding of small-molecule modulator binding to P2X receptors, and use this data in conjunction with molecular docking experiments to develop novel hypotheses for the involvement of the dorsal fin region in altered agonist potency at P2X7 receptors, and how the P2X7-selective agonist BzATP and the P2X4 positive allosteric modulator ivermectin bind to their respective receptor subtypes. We first focus on the currently available crystal structures of P2X receptors, highlighting which portions of the receptor they cover, and how they are related by amino-acid sequence identity. We briefly discuss the conformational changes induced by ATP binding, before analyzing the orthosteric binding sites in zfP2X4, human P2X3, gulf coast tick P2X, and chicken P2X7, and the allosteric binding site in panda P2X7. Finally, we build upon published data from structure-function experiments, using molecular docking simulations to hypothesize how the P2X7 agonist BzATP may be able to bind more effectively than ATP at the rat isoform of this receptor by interacting with Lys-127 adjacent to the ATP binding pocket, and how ivermectin may stabilize the open state of rat P2X4 receptors by binding between the first and second transmembrane domains.

## Modulator binding in P2X receptor crystal structures

### Analysis of solved P2X structures

**Figure 1:**
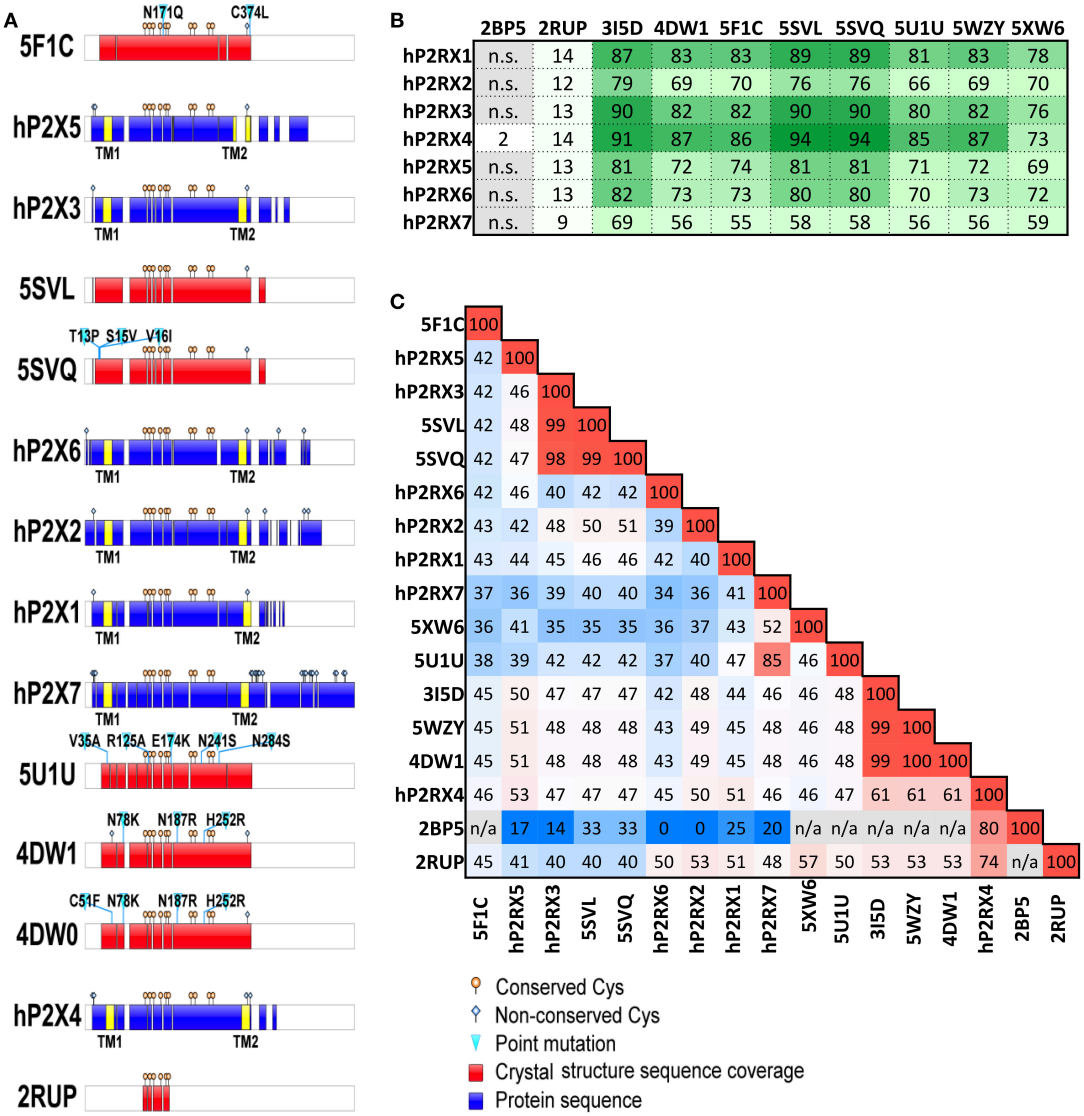
Published P2X receptor constructs; sequence coverage and amino-acid identity. **(A)** Graphical representation of coverage of human P2Xs sequence by crystal structures showing aligned domains. Transmembrane domains (TM) highlighted in yellow; white sections represent gaps. 4DW0 was represented separately to highlight the presence and location of an additional mutation compared to 4DW1. The graphical representation of 5XW6 (chicken P2X7 structure) is not shown. **(B)** Percentage coverage of human P2X receptor sequences by each crystal structure, calculated using BlastP. **(C)** Amino-acid identity matrix of human P2X receptor sequences and sequences listed in Table 1 (values in percentage; blue for lowest identity, red for highest identity, n/a for non-alignable sequences). Sequences of human P2X (hP2X) receptors were retrieved from UniProt database (Uniprot IDs: P51575, hP2RX1; Q9UBL9, hP2RX2; P56373, hP2RX3; Q99571, hP2RX4; Q93086, hP2RX5; O15547, hP2RX6; Q99572, hP2RX7) and aligned with PDB sequences using ClustalW. Sequences with more than 99.5% identity were grouped together under a single PDB ID for clarity (e.g., 5SVL with 5SVM and 5SVP; 5SVQ with 5SVJ, 5SVK, 5SVR, 5SVT and 5SVS; 4DW1 with 4DW0 and 3H9V; 5U1U with 5U1L, 5U1V, 5U1W, 5U1X, 5U1Y and 5U2H).

To date, high-resolution structural data has been obtained for a total of six different P2X receptor constructs, including an NMR structure of the isolated head domain of rat P2X4 (Igawa et al., 2015), and crystal structures of zfP2X4 (Kawate et al., 2009; Hattori and Gouaux, 2012; Kasuya et al., 2017a), Gulf Coast tick P2X (Kasuya et al., 2016), human P2X3 (Mansoor et al., 2016), panda P2X7 (Karasawa and Kawate, 2016), and chicken P2X7 (Kasuya et al., 2017b). Each available entry in the Protein Data Bank is listed in Table 1, along with the state in which the crystal structure was determined and its reported resolution. It is important to note that none of these structures represents the native, full-length receptor; the regions of protein sequence encompassed by each representative structure are shown in comparison to the full-length human P2X receptors in Figure 1A, and the percentage sequence coverage compared to each human P2X receptor shown in Figure 1B. All “crystal constructs” are truncated to some degree at both the N- and C-termini (and indeed the N- and C-termini of the crystal constructs are often not observed in the crystal structures due to their inherent flexibility), and all (apart from human P2X3) bear point mutations. The effects of these truncations and/or mutations on protein function are significant in some cases; the original zfP2X4 *apo*-state crystal structure was significantly impaired in ion channel function (Kawate et al., 2009; Young, 2010), the Gulf Coast tick P2X construct, while still able to bind ATP, did not form a functional ion channel (Kasuya et al., 2016), and the chicken P2X7 construct, while still able to bind TNP-ATP, was also non-functional (Kasuya et al., 2017b). However, later zfP2X4 constructs (used to elucidate new *apo*-state and ATP-bound state structures) displayed channel function approaching that of the native receptor (Hattori and Gouaux, 2012), and human P2X3 constructs were fully functional compared to wild-type receptors (Mansoor et al., 2016). The amino-acid sequence identities between the full-length human P2X receptors and the crystal constructs (Figure 1C) demonstrate that these structures are very useful as templates for homology modeling with the human receptors that lack crystal structures, due to the high degree of similarity between them.

**Table 1:** List of P2X receptor structures (PDB codes) solved to date.

**PDB ID**	**Resolution (Å)**	**P2X subtype (Species)**	**State (ligand)**
2BP5	2.8	P2X4 (rat)	n/a
2RUP	^*^	P2X4 (rat)	n/a
3H9V	3.1	P2X4 (zebrafish)	Apo
3I5D	3.46	P2X4 (zebrafish)	Apo
4DW0	2.9	P2X4 (zebrafish)	Apo
4DW1	2.8	P2X4 (zebrafish)	Bound (ATP)
5F1C	2.9	P2X (Gulf Coast tick)	Bound (ATP)
5SVJ	2.98	P2X3 (human)	Apo
5SVK	2.77	P2X3 (human)	Bound (ATP); open
5SVL	2.9	P2X3 (human)	Bound (ATP); desensitized
5SVM	3.09	P2X3 (human)	Bound (2-methylthio-ATP); desensitized
5SVP	3.3	P2X3 (human)	Bound (2-methylthio-ATP); desensitzed
5SVQ	3.25	P2X3 (human)	Bound (TNP-ATP)
5SVR	3.13	P2X3 (human)	Bound (A-317491)
5SVS	4.03	P2X3 (human)	Apo
5SVT	3.79	P2X3 (human)	apo
5U1L	3.4	P2X7 (giant panda)	Apo
5U1U	3.6	P2X7 (giant panda)	Bound (A740003)
5U1V	3.4	P2X7 (giant panda)	Bound (A804598)
5U1W	3.5	P2X7 (giant panda)	Bound (AZ10606120)
5U1X	3.2	P2X7 (giant panda)	Bound (JNJ47965567)
5U1Y	3.3	P2X7 (giant panda)	Bound (GW791343)
5U2H	3.9	P2X7 (giant panda)	Bound (ATP, A804598)
5WZY	2.8	P2X4 (zebrafish)	Bound (CTP)
5XW6	3.1	P2X7 (chicken)	Bound (TNP-ATP)

All structures were solved using X-ray diffraction except 2RUP (marked with ^*^), which was obtained using NMR. n/a refers to sequences with <60 amino acids.

* *NMR structure*.

### The conformational change induced by ATP binding

**Figure 2:**
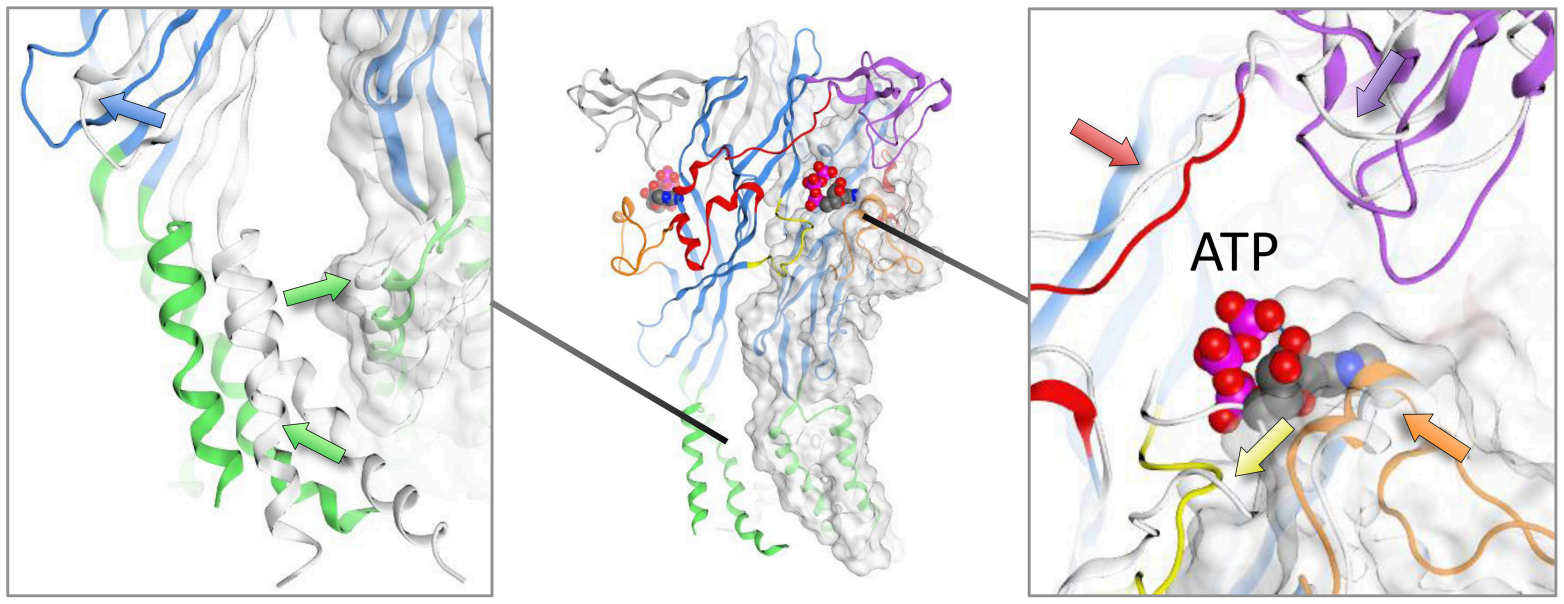
Conformational changes observed in zebrafish crystal structure upon ATP binding. Structures shown are zfP2X4; PDB IDs: 4DW0 (*apo*), and 4DW1 (ATP-bound). Arrows show the trajectories of the backbone rearrangement. The *apo*-structure is presented in white ribbons while the ATP-bound structure follows the canonical domain coloring scheme (green, transmembrane domains; blue, lower and upper body; purple, head domain; orange, dorsal fin; red, right flipper; yellow, left flipper). Detail of transmembrane region and the ATP-binding pocket are shown in the left and right panels, respectively.

The conformational changes induced by the binding of ATP (and the partial agonist CTP) have been captured in the crystal structures of zfP2X4 (Hattori and Gouaux, 2012; Kasuya et al., 2017a) and human P2X3 (Mansoor et al., 2016). These changes are vital for the agonist-induced gating of the cation channel, and while not central to the focus of this article, are discussed briefly here. For a detailed treatment of the structural basis for the activation of P2X receptors see (Kawate, 2017). Using the *apo*- and ATP-bound zfP2X4 crystal structures as a guide (Hattori and Gouaux, 2012), ATP binding into its pocket in a cleft between subunits (Figure 2, center panel) induces movements in the extracellular domain of the receptor (Figure 2, right panel), leading to the upward movement of the dorsal fin domain and the closure of the head region around the ATP binding site, and the downward movement of the left flipper domain. This in turn leads to movement in the lower body of the extracellular domain, which has the effect of moving the second transmembrane domain (which lines the channel) relative to the first transmembrane domain (Figure 2, left panel), opening the channel in a manner similar to a camera iris. In the recent ATP-bound human P2X3 crystal structure (Mansoor et al., 2016), the center of the second transmembrane domain is observed to transition from an α-helix to a 3_10_ helix, which may stabilize channel opening.

### The orthosteric binding site

**Figure 3:**
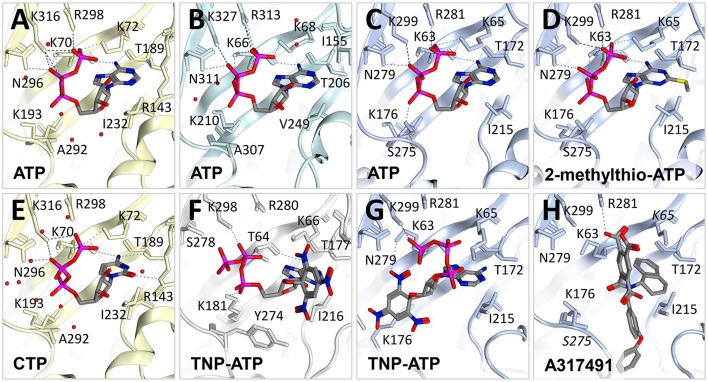
Molecular determinants of orthosteric binding. Crystal structures of zebrafish P2X4 (light yellow ribbon) bound to **(A)** ATP (PDB ID: 4DW1) and **(E)** CTP (5WZY). Water molecules are shown as red dots. **(B)** Gulf coast tick crystal structure (in light cyan ribbon) in the presence of ATP (5F1C). Human P2X3 crystal structures (light blue ribbon) bound to **(C)** ATP (5SVK), **(D)** 2-methylthio-ATP (5SVP), **(G)** TNP-ATP (5SVQ) and **(H)** A317491 (5SVR). **(F)** Crystal structure of chicken P2X7 (gray ribbon) in the presence of TNP-ATP (5XW6). H-bond interactions are displayed with dotted lines. Only residues in close proximity to the ligand are labeled. Note that crystal structure data relative to the ATP binding site is incomplete in **(G)** where the loop containing S275 is missing from the PDB file and in **(F)** where K65 and S275 (labeled in italics) have missing atoms. Figures were made using Molecular Operating Environment (MOE).

Prior to the availability of P2X receptor crystal structures complexed with ATP, several conserved amino-acids critical for ligand binding were identified in a series of key structure-function experiments (reviewed in Browne et al., 2010; Young, 2010), and these residues were demonstrated to line a pocket between receptor subunits in the extracellular domain in the first P2X crystal structure, *apo*-zfP2X4 (Kawate et al., 2009). ATP-bound structures are now available for zfP2X4 (Hattori and Gouaux, 2012), Gulf Coast tick P2X (Kasuya et al., 2016), and human P2X3 [also obtained in the presence of the agonist 2-methylthio-ATP (Mansoor et al., 2016) (Figures 3A–D); they display near-identical U-shaped conformations. Of critical importance for agonist activity are the polar interactions with the γ-phosphate and the adenine ring; P2X receptors are not activated by ADP [although tethering of ligands to one binding site of P2X7 has been shown to alter the specificity of other binding sites in the same receptor, enabling further activation by ADP and CTP (Browne and North, 2013)]. P2X receptors display selectivity for purines over pyrimidines, but CTP is a partial agonist at some P2X receptors, including zfP2X4 (Kasuya et al., 2017a). In the crystal structure of CTP-bound zfP2X4 (Figure 3E), a hydrogen bond is observed between the N-4 atom of cytosine and the main chain of Thr-189 (zfP2X4 numbering), and an additional hydrogen bond is formed between the O-2 atom of the cytosine ring and the sidechain of Arg-143. This enables the CTP to adopt a similar conformation to that of ATP, even though the pyrimidine ring is smaller. Another point of note in all 5 agonist-bound structures is the close proximity between the γ-phosphate oxygen and the 2′-hydroxyl group of the ribose, which may give rise to hydrogen-bond formation. This hydrogen bond may be very important for maintaining the stability of the ATP molecule in the P2X receptor orthosteric binding site, as suggested by recent molecular dynamics simulations and the observation that 2′-deoxy-ATP is a poor agonist at human P2X1 receptors in contrast to 3′-deoxy-ATP (Fryatt et al., 2016). Structures of human P2X3 determined in complex with the orthosteric antagonists TNP-ATP (Figure 3G) and A-317491 (Figure 3H) (Mansoor et al., 2016), and chicken P2X7 in complex with TNP-ATP (Figure 3F) (Kasuya et al., 2017b) give a fascinating insight into the molecular basis for competitive antagonism at these receptors. Somewhat surprisingly, when bound to human P2X3 TNP-ATP adopts a strikingly different conformation to that of ATP; although the γ-phosphate is in a broadly similar position, the molecule adopts a Y-shaped conformation, making a hydrophobic interaction deep in the cleft between subunits that ATP does not access (Figure 3G). This Y-shaped conformation and deeper binding is also observed for A-317419 (Figure 3H), and the effect is to prevent the upward movement of the dorsal fin domain, precluding the closure of the binding cleft that is a prerequisite for channel opening (Mansoor et al., 2016). In chicken P2X7, the TNP-ATP conformation is markedly different to that observed in human P2X3 (Figure 3F). The adenine ring and ribose adopt similar conformations to that observed for ATP in other P2X receptor crystal structure, but the position of the TNP moiety is altered (contacting the head and dorsal fin domains, rather than buried in the body domain as observed in human P2X3), and the phosphate chains display an extended conformation (Kasuya et al., 2017b). Kasuya et al. used molecular dynamics simulations to conclude that, while TNP-ATP binding to chicken P2X7 induces initial conformational changes similar to those which might be expected for ATP, the TNP moiety prevents downward movement of the head domain induced by cleft closure of the ATP binding pocket and consequently prevents activation of the receptor (Kasuya et al., 2017b). We propose that the observation of different binding conformations for TNP-ATP in P2X3 and P2X7 subtypes may well reflect the 1,000-fold difference in potency of TNP-ATP at P2X3 and P2X7 receptors [IC_50_ ~ 1 nM for P2X3 (North, 2002) and 3.55 μM for chicken P2X7 (Kasuya et al., 2017b)], suggesting that a competitive antagonist which can bind deeper into the cleft between subunits may well be significantly more potent than one which prevents movement of the head domain.

### An allosteric binding site in P2X7

**Figure 4:**
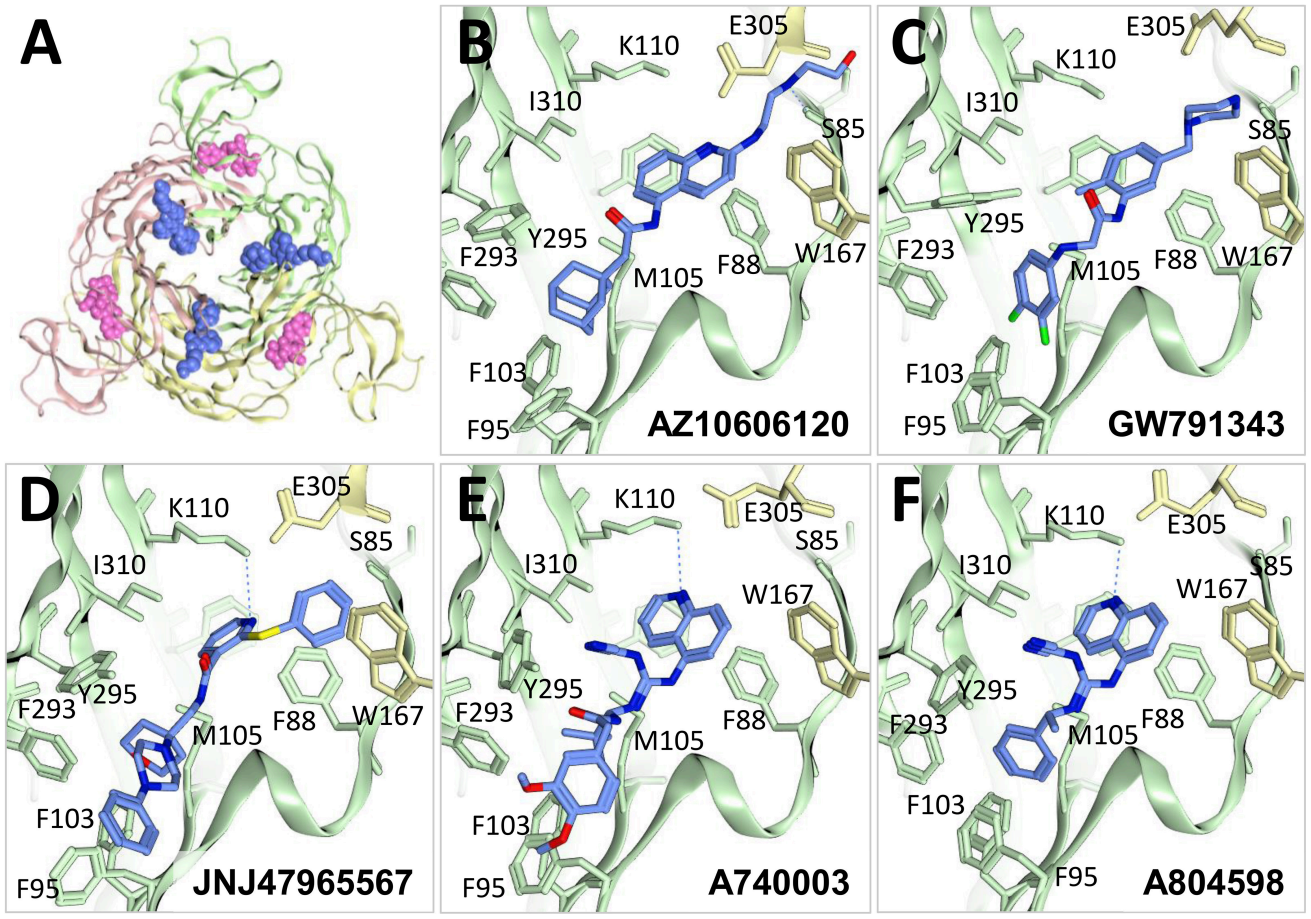
Allosteric antagonists bound to P2X7. **(A)** Top view of giant panda P2X7 crystal structure (PDB ID: 5U1W) bound to AZ10606120 antagonist (blue). ATP (pink) was superposed to highlight the difference between the location of the allosteric pocket and the ATP-binding site. Crystal structures of giant panda bound to **(B)** AZ10606120 (5U1W), **(C)** GW791343 (5U1Y), **(D)** JNJ47965567 (5U1X), **(E)** A740003 (5U1U) and **(F)** A804598 (5U1V) showing residues near the allosteric antagonist colored according to **(A)**. F108 is not labeled for clarity. Figures were made using MOE.

The P2X7 receptor, with its wide-ranging proposed physiological roles in inflammatory disease, cancer, neurological disorders (Bartlett et al., 2014; Roger et al., 2015; Pevarello et al., 2017) and, most recently, metabolic disease (Arguin et al., 2017), has been a focus within the pharmaceutical industry and several potent and selective P2X7 antagonists have been developed (Park and Kim, 2017). Many of these were thought to be orthosteric, but painstaking and elegant recent work by Alsopp et al, using structure-function and molecular modeling (Allsopp et al., 2017), and by Karasawa and Kawate, using functional assay and crystallization of giant panda P2X7 in complex with a series of P2X7 antagonists (Karasawa and Kawate, 2016) has clearly demonstrated that these compounds bind to an allosteric site in the extracellular domain distinct from the ATP binding pocket (Figure 4A). Crystals were obtained in complex with AZ10606120, GW791343, JNJ47965567, A740003, and A804598 (Figures 4B–F). Comparison of the binding of these compounds shows either an elongated conformation which appears to extend across subunits (AZ10606120, GW791343, JNJ47965567), or a more compact conformation making a polar interaction between A740003 and A804598 and the side-chain of Lys-110. In all cases extensive hydrophobic interactions are observed in a pocket delineated by Phe-95, Phe-103, Met-105, Phe-293, and Tyr-295 (panda P2X7 numbering). The discovery of this new allosteric binding pocket in P2X7 (and the availability of the crystal structure as a modeling template) should greatly facilitate the structure-aided design of new allosteric P2X7 antagonists; it may also be possible to exploit this pocket in other receptor subtypes to develop antagonists with increased subtype selectivity.

### Comparison of the dorsal fin loops in P2X3, P2X4, and P2X7

**Figure 5:**
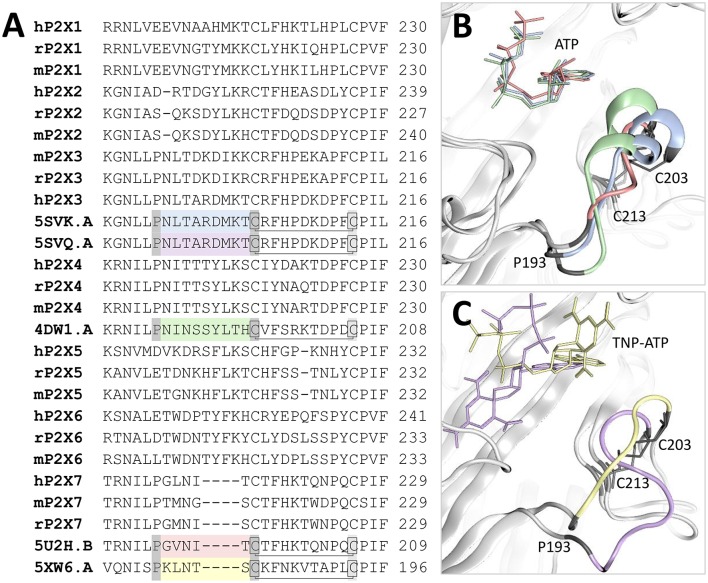
Comparison of the dorsal fin loop structure in P2X3, P2X4, and P2X7. **(A)** Sequence alignment of mouse (m), rat (r), and human (h) P2X receptors with crystal structures bound to either ATP (hP2X3, 5SVK.A; zfP2X4, 4DW1.A; panda P2X7, 5U2H.B) or TNP-ATP (hP2X3, 5SVQ.A; chick P2X7, 5XW6.A). For each crystal structure the dorsal fin region is shown in a different color corresponding to those displayed in **(B,C)** (blue for hP2X3-ATP, purple for hP2X3-TNP-ATP, green for zfP2X4-ATP, pink for panda P2X7-ATP and yellow for chick P2X7-TNP-ATP), and the amino-acid residues at the loop boundaries are shown in dark gray boxes. The conserved disulfide bond is shown with a line. **(B)** Comparison of the ATP-bound structures of hP2X3, zfP2X4, and panda P2X7 with the dorsal fin loop shown in blue, purple, and pink respectively. The shorter dorsal fin region of P2X7 leads to a significantly altered loop conformation which may affect the ability of the receptor to effect conformational change following ATP binding. **(C)** Comparison of the TNP-ATP-bound structures of hP2X3 and chick P2X7 with the dorsal fin loop shown in purple and yellow respectively. The disulfide bond is indicated with gray lines. The dorsal fin loop conformation of hP2X3 is significantly altered compared to that observed in the ATP-bound structure, suggesting that the conformational change has not taken place.

P2X receptor subtypes display substantial differences in relative ATP potency. For example, ATP EC_50_ values for human P2X3, human P2X4, and human P2X7 have been reported as 0.13, 7.4, and 720 μM (in low divalent cation-containing solution), respectively (Garcia-Guzman et al., 1997; Neelands et al., 2003; Stokes et al., 2006). Particularly striking is the >1,000-fold lower ATP potency observed at P2X7 receptors compared to other subtypes. Lower potency could be either due to reduced agonist affinity, or reduced ability of the agonist to effect a response (efficacy, here considered as the ability of the agonist to induce a conformational change in the receptor). Because the residues important for binding ATP in P2X receptors and the overall shape of the ATP binding pocket are well-conserved across subtypes, we hypothesize that lower ATP potency observed at P2X7 receptors may result from reduced efficacy. There are a number of regions of sequence difference between P2X7 and the other subtypes which could be responsible for differences in efficacy, but multiple sequence alignment of receptor subtypes shows a clear candidate; the dorsal fin loop (Figure 5A). This loop moves upward on ATP binding (Hattori and Gouaux, 2012; Mansoor et al., 2016), and P2X7 subtypes bear a 4 amino-acid deletion in this region, substantially changing its conformation in P2X7 compared to P2X3 and P2X4, both in complex with ATP (Figure 5B) and TNP-ATP (Figure 5C). It is interesting to note that while the dorsal fin conformation in human P2X3 bound to ATP and TNP-ATP is markedly different (compare Figures 5B,C), suggesting a lack of movement of the dorsal fin in the antagonist-bound structure, the conformation of this region in P2X7 is less affected. One reason for this may be that, in chick P2X7, TNP-ATP adopts a similar conformation to ATP, and so a conformational change in the dorsal fin region has taken place in this structure (unfortunately the corresponding *apo*-structure is not available for comparison). An alternative hypothesis is that the shorter dorsal fin loop in P2X7 does not undergo the extensive conformational change seen in P2X3, effectively limiting the degree of conformational change that occurs in P2X7 upon ATP binding, and thus lowering the efficacy of ATP. One way to test this hypothesis would be to replace the dorsal fin loop region in P2X7 with that of another subtype (and *vice versa*) and testing the effects of this substitution on ATP EC_50_. It should be noted that the conformation of the dorsal fin region of each crystal structure may be affected by crystal contacts in the unit cell of the crystals used for structure determination, and also that these regions of the protein are likely to be dynamic and mobile, and may be capable of adopting a range of conformations in either *apo*- or ligand-bound states.

## Modeling modulator binding

### The semi-selective P2X7 agonist BzATP

**Figure 6:**
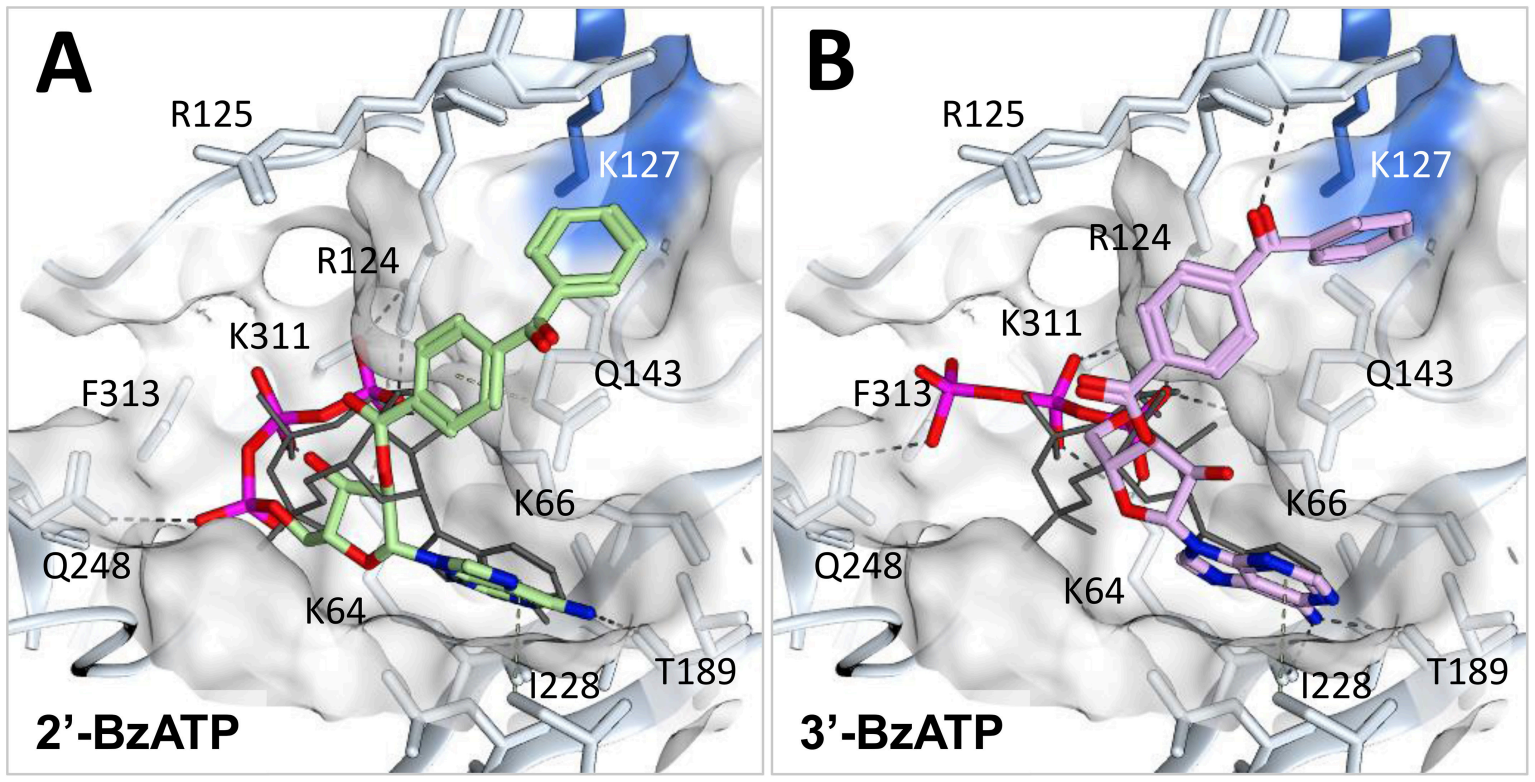
Docking of BzATP into mouse P2X7 A127K. A mouse P2X7 homology model (Uniprot sequence: Q9Z1M0, which was modified to obtain the A127K mutation) was generated using Molecular Operating Environment (MOE) software (MOE 2014.10; Chemical Computing Group ULC, 1010 Sherbooke St. West, Suite #910, Montreal, QC, Canada, H3A 2R7, 2018). The sequences of mouse P2X7 receptor and 5U2H.pdb [chain B; retrieved from RCSB Protein Data Bank (http://www.rcsb.org)] were aligned and the trimeric system was prepared by adding hydrogens and calculating partial charges according to the AMBER10:EHT forcefield. The MOE Built-in homology modeling tool was then used with default settings to make the model, maintaining ATP molecules in the environment and enabling the induced-fit option. As per default settings, the final model was refined after the generation of 10 intermediate models. Schrödinger Suite was employed for all the docking simulations and their preprocessing. In brief, the model was prepared, checked and parameterized with Protein Preparation Wizard (Schrödinger Suite 2015-1, Protein Preparation Wizard; Epik, Schrödinger, LLC, New York, NY; Impact, Schrödinger, LLC, New York, NY; Prime, Schrödinger, LLC, New York, NY). The two isomers of BzATP were built using the MOE Builder tool then preprocessed with LigPrep (Schrödinger, LLC, New York, NY) employing the OPLS_2005 force field and Epik to generate ionization states at pH 7.0 ± 2.0. Docking of the ligands in the competitive binding site was performed using Glide extra precision (release 2015-1, Glide, Schrödinger, LLC, New York, NY) within a box of 38Å^3^, using superposed ATP as the centroid of the box. **(A)** 2′-BzATP (green) and **(B)** 3′-BzATP (lilac) compared to the superposed ATP crystal conformation (dark gray lines). The surface of the pocket is represented in white, the surface of the shallow pocket where the Lys-127 residue is located near the benzoyl group is represented in blue. H-bond interactions are shown in dark gray dotted lines, hydrophobic interactions in green dotted lines.

BzATP is a synthetic ATP derivative with a benzoylbenzoyl moiety linked to either the 2′ or the 3′ oxygen of ribose. Commercially available BzATP preparations are a mixture of the two isomers and to our knowledge, it is not known whether just one or both isomers have activity at P2X7 receptors. Some initial studies describe the application of the 3′ isomer, but this may be because the original paper describing BzATP synthesis reported it as the 3′ isomer (Williams and Coleman, 1982). However, later synthesis and characterization using similar methods reported a mixture of 3′ and 2′ isomers in a 60:40 ratio (Mahmood et al., 1987). BzATP is more potent than ATP at the P2X7 receptor, and this attribute can be used as pharmacological evidence to support functional expression of P2X7 receptors. However, it is important to note that BzATP is also a full agonist at P2Y receptors (Boyer and Harden, 1989), a partial agonist at P2X4 receptors (Bowler et al., 2003), and an agonist at P2X1, P2X2, and P2X5 receptors (Evans et al., 1995; Bo et al., 2003), meaning that claims of physiological roles for P2X7 receptors based on BzATP-induced responses must be treated with some caution. However, at P2X7 receptors, BzATP is significantly more potent than ATP (~30-fold at rat P2X7 compared to 4-fold at mouse P2X7, Young et al., 2007) and elicits larger whole-cell responses (Surprenant et al., 1996). It has previously been demonstrated that BzATP potency in rat P2X7 is governed by Lys-127 (the mutation A127K in mouse P2X7 significantly increases BzATP potency relative to ATP), and suggested that the side-chain of this residue might be able to form a π-cation interaction with one of the benzene rings of BzATP (Young et al., 2007). To test this hypothesis, we docked both isomers of BzATP into the ATP binding site of a molecular model of mouse P2X7 bearing the A127K mutation (Figure 6). Docking of 2′-BzATP reproduced a strikingly similar conformation to that of ATP (Figure 6A). Approximate positions of the phosphate chains and adenine moiety were preserved; however the ribose of BzATP was translated relative to ATP, presumably to accommodate the benzoylbenzoyl moiety. Docking of 3′-BzATP (Figure 6B) was less satisfactory; although the positions of the adenine and ribose were well-conserved, the position of the critical γ-phosphate was not. Strikingly, in both conformations, the benzene ring most distant from the ribose was positioned very near to Lys-127, suggesting that formation of a π-cation interaction is plausible. In summary, our docking suggests not only that the original molecular hypothesis for enhanced BzATP potency at rat P2X7 (Young et al., 2007) may be correct, but also that the 2′ isomer of BzATP should possess greater activity at P2X7 receptors than the 3′ isomer. Testing this hypothesis would require the selective purification of 2′- and 3′-BzATP isomers.

### The P2X4 positive allosteric modulator ivermectin

**Figure 7:**
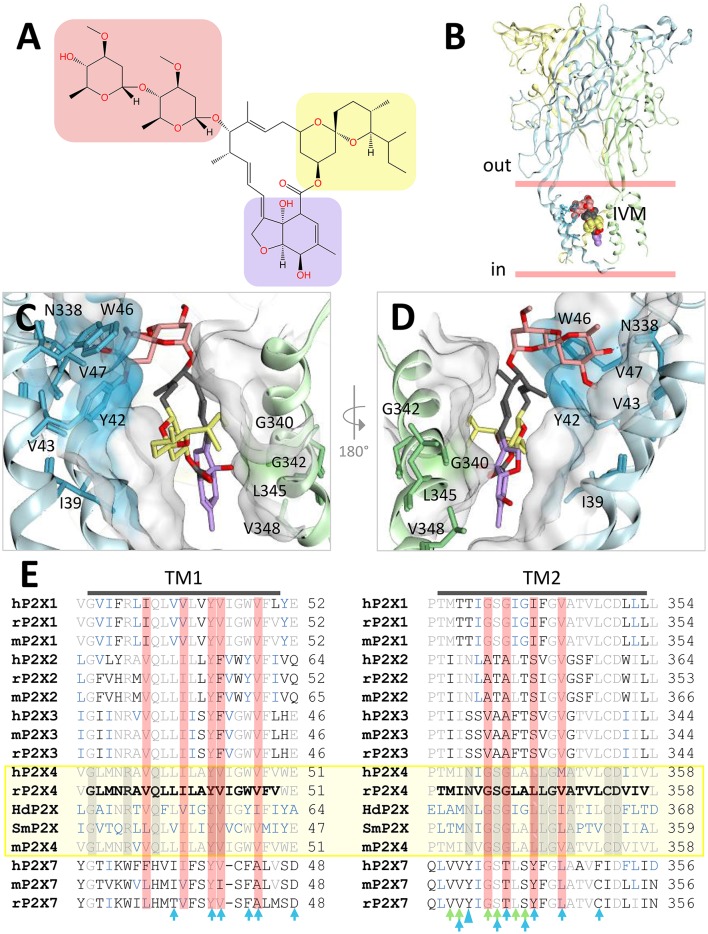
Docking of ivermectin (IVM) in the predicted allosteric binding site of the rat P2X4 homology model. A rat P2X4 homology model (Uniprot sequence: P51577) was built using MOE with 4DW1 structure as a template and prepared for docking with Protein Preparation Wizard (see Figure 6). Docking was performed within a box of 35Å^3^ located at the level of the transmembrane domain using the coordinates of a previously identified pocket [with Site Finder (MOE)] as the centroid of the box. **(A)** Chemical structure of ivermectin B1a, highlighting the dioleandrose (disaccharide) (salmon), spiroketal (yellow), and hydrogenated benzofuran (purple). **(B)** Location of the predicted IVM binding site in rat P2X4. The membrane bilayer is delimited by red lines. IVM atoms are colored according to **(A)** while the receptor is represented in ribbons, each color representing a different subunit. **(C)** Detail of IVM conformation docked between two subunits, using the same color scheme as in **(A)**. The residues close to the IVM pose and identified to be important in IVM activity (see text) are shown and colored according to the receptor subunit. The surface of the receptor is shown in white; colored areas correspond to the highlighted residues. **(D)** 180° rotation of the IVM pose displayed in **(C)**. The third subunit (yellow in **B**) is omitted for clarity. **(E)** Sequence alignment of the transmembrane (TM) domains of human (h), rat (r), mouse (m), *Schistosoma mansoni* (Sm), and *Hypsibius dujardini* (Hd) P2X receptor sequences. Amino-acids identical to those found in rP2X4 are colored in gray. Receptors sensitive to IVM are shown in a yellow box, and residues conserved across all subtypes are encapsulated in gray boxes. Residues colored blue are those which are conserved in P2X receptor subtypes where IVM is active. Residues in red boxes are those identified by Silberberg et al. to significantly reduce IVM activity when mutated to tryptophan (Silberberg et al., 2007). Blue and green arrows indicate the amino-acid residues within a distance of 4.5Å of IVM in our rP2X4-IVM dock; arrows are colored according to the receptor subunit involved in the interaction (same color scheme as **A**). The large blue arrow head indicates Asn-338, which in our docking simulation forms an H-bond with the oleandrose moiety of IVM.

P2X4 receptors play an important role in the central nervous system (CNS) in the modulation of neuroinflammation and neuropathic pain, and are important CNS drug targets (Stokes et al., 2017). Ivermectin (Figure 7A) is an anti-helminthic drug used in veterinary medicine, and in humans to treat river blindness (onchocerciasis) (Zemkova et al., 2014). It kills nematodes by activating glutamate-gated chloride channels and has been shown to affect the activity of several other ligand-gated ion channels, including glycine receptors, γ-amino butyric acid (GABA) receptors, nicotinic acetylcholine receptors, and P2X4 receptors (Zemkova et al., 2014; Chen and Kubo, 2017). The mode of action of ivermectin on the cys-loop ligand-gated ion channel family has been structurally characterized; ivermectin is known to bind between transmembrane domains, stabilizing the channel open state (Hibbs and Gouaux, 2011). In this way ivermectin acts as a positive allosteric modulator. It is currently unknown how ivermectin binds to P2X4 receptors, but it is known that ivermectin exerts two effects on P2X4; it causes an increase in maximum current amplitude (this effect occurs at relatively low concentrations of ivermectin and has an EC_50_ of 0.25 μM), and at it slows receptor deactivation by stabilizing the open conformation of the channel (this effect occurs at relatively high concentrations of ivermectin and has an EC_50_ of 2 μM) (Priel and Silberberg, 2004). The dual effects of ivermectin potentially suggest two (or more) binding modes, but it is plausible to assume that it may stabilize the open conformation of P2X4 by a similar mechanism to that observed in cys-loop receptors. Indeed, a series of mutagenesis and structure-function experiments have been conducted in order to determine amino-acid residues important for the effects of ivermectin at P2X4 receptors (Silberberg et al., 2007; Samways et al., 2012; Tvrdonova et al., 2014), reviewed in (Chen and Kubo, 2017) and the majority of these residues are clustered toward the upper (extracellular side of the membrane) and central portions of the first transmembrane domain, and the central and lower portions of the second transmembrane domain (Figure 7B). Using these amino-acids as a reference, we were able to dock ivermectin B1a into a molecular model of rat P2X4 between the first and second transmembrane domains from adjacent subunits (Figure 7B). In our docking simulation the ivermectin molecule is positioned with the hydrogenated benzofuran moiety toward the intracellular face of the membrane, forming hydrophobic interactions with Leu-345 and Val-348 near the bottom of the second transmembrane domain of one subunit (colored blue in Figures 7C,D). The disaccharide moiety (dioleandrose) is positioned near the extracellular face, forming hydrophobic interactions with Tyr-42, Trp-46, and Val-47 at the top of the first transmembrane domain, and a hydrogen bond between the hydroxyl group of the distal oleandrose and the oxygen atom of the side-chain of Asn-338 near the top of the second transmembrane domain of the adjacent subunit (colored green in Figures 7C,D). This means that ivermectin is interacting with the bottom of the second transmembrane domain in one subunit, and the top of both the first and second transmembrane domains in the adjacent subunit, which would have the effect of stabilizing the channel in the open state following activation by ATP. It is important to note that this conformation does not preclude ivermectin from binding to the other two equivalent binding sites in the trimer.

In order to understand why ivermectin is capable of potentiating current responses at some P2X receptor subtypes but not others, we aligned the transmembrane domain sequences of human, rat, and mouse P2X1, P2X1, P2X3, P2X4, and P2X7, along with those of *Schistosoma mansoni* P2X (Agboh et al., 2004) and *Hypsibius dujardini* P2X (Bavan et al., 2009), also shown to be sensitive to ivermectin (Figure 7E). The ivermectin-sensitive receptor subtypes are grouped together in a yellow box (Figure 7E). It should be noted that ivermectin sensitivity has been reported in human P2X7 (but not mouse P2X7) in whole-cell patch clap experiments on transfected human cells (Norenberg et al., 2012); however, a recent study on human P2X7 expressed in *Xenopus* oocytes observed no potentiation (Schneider et al., 2017). In Figure 7E, amino-acid residues identical to rat P2X4 are colored in gray, amino-acids conserved across all subtypes are in gray boxes, and amino-acids conserved in subtypes where ivermectin is active are shown in blue. Red boxes highlight the amino-acid residues where substitution with tryptophan significantly reduced ivermectin activity in rat P2X4 (Silberberg et al., 2007). Of the nine amino-acids implicated in ivermectin action by tryptophan mutagenesis, seven are within 4.5 Å of the ivermectin molecule in our dock (green and blue arrows indicate residues in close proximity in Figure 7E), showing a good correlation between our docking simulation and experimental data. In our docking simulation we observed a hydrogen bond between the sidechain of Asn-338 and the oleandrose moiety of ivermectin (Figures 7C,D, large blue arrowhead in Figure 7E). Interestingly, the rat P2X4 mutant N338W retained ivermectin sensitivity (Silberberg et al., 2007). We explored this by modeling ivermectin binding to the rat P2X4 N338W mutant, finding that ivermectin was still able to make an H-arene interaction with the tryptophan (data not shown). We hypothesize that mutating this residue to one incapable of forming H-bonds (such as Ile or Leu) should significantly impair the ability of ivermectin to potentiate current responses at rat P2X4. Analysis of sequence conservation across the transmembrane domains suggests that the P2X1 subtype is most similar to the ivermectin-sensitive subtypes. While it may not be possible to confer ivermectin sensitivity to another subtype with one point mutation, we suggest that a combination of the mutations F33N, T333I, T334N, I341L, and F342L in human P2X1 may confer ivermectin sensitivity to this receptor subtype.

In summary, our docking simulation explains the ability of ivermectin to stabilize the open state of P2X4 receptors, and hints at the amino-acid residues important for the subtype-specific effects of ivermectin; furthermore, our docking should permit the rational design of smaller lipophilic molecules which specifically target sub-regions of the putative ivermectin binding site in P2X4. It is important to note that Glu-51, located in the lateral ion-access portal just above the transmembrane domains and conserved in P2X1 and P2X3 receptors (shown in Figure 7E), has also been implicated in the action of ivermectin (Samways et al., 2012). We did not observe an interaction between ivermectin and Glu-51 in our docking simulation; it is possible that the region including and surrounding Glu-51 may contribute to a second ivermectin binding site, which may explain the dual actions of ivermectin on P2X4 receptors. Finally, we note that, as our manuscript was at the proof stage, a molecular dock of P2X4 bound to ivermectin was published (Latapiat et al., 2017) which appears to adopt a similar conformation to that shown in our docking simulation.

## Concluding remarks

This article demonstrates how the growing collection of P2X receptor crystal structures, from different species and subtypes, in complex with orthosteric agonists and both orthosteric and allosteric antagonists, has not only transformed our understanding of how small molecule modulators bind to and influence P2X receptor function, but also has enabled us to effectively model novel binding conformations for other ligands based on the available molecular evidence. We have been able to compare the binding of ATP at zfP2X4, Gulf Coast tick P2X and human P2X3, and to analyze the molecular basis for competitive and non-competitive antagonism at human P2X3 and panda and chicken P2X7. The orthosteric antagonist binding site in P2X7 may well be a valid target for future structure-based drug design in other receptor subtypes in order to develop subtype-selective antagonists. We have analyzed the difference in conformation of the dorsal fin loop between P2X7 and P2X3 and P2X4, hypothesizing that the shorter loop in P2X7 may affect the degree of conformational change induced by ATP binding, and hence the efficacy of the agonist. Using molecular modeling and docking experiments, we have been able to propose plausible and testable binding conformations for the agonist BzATP at P2X7, and the positive allosteric modulator ivermectin at rat P2X4, hypothesizing that the 2′ isomer of BzATP should be more potent than the 3′ isomer, and outlining a series of mutations which may confer ivermectin sensitivity onto P2X1 receptors. While a comprehensive structural basis for subtype-specific differences in agonist potency is still lacking, it is to be hoped that structures of P2X1 and P2X2 receptor subtypes, where the majority of structure-function studies have been conducted, will be solved in the near future, giving new insights into ligand binding and a better context to previously published data.

## Author contributions

GP and AB: Performed and analyzed ligand docking experiments; GP: Prepared the Figures, MY: Wrote the manuscript; GP, AB, and MY: Edited the manuscript.

### Conflict of interest statement

The authors declare that the research was conducted in the absence of any commercial or financial relationships that could be construed as a potential conflict of interest.
